# CD1b Tetramers Broadly Detect T Cells That Correlate With Mycobacterial Exposure but Not Tuberculosis Disease State

**DOI:** 10.3389/fimmu.2020.00199

**Published:** 2020-02-14

**Authors:** Kattya Lopez, Sarah K. Iwany, Sara Suliman, Josephine F. Reijneveld, Tonatiuh A. Ocampo, Judith Jimenez, Roger Calderon, Leonid Lecca, Megan B. Murray, D. Branch Moody, Ildiko Van Rhijn

**Affiliations:** ^1^Socios En Salud, Lima, Peru; ^2^Division of Rheumatology, Inflammation, and Immunity, Brigham and Women's Hospital and Harvard Medical School, Boston, MA, United States; ^3^Department of Infectious Diseases and Immunology, Faculty of Veterinary Medicine, Utrecht University, Utrecht, Netherlands; ^4^Division of Global Health Equity, Department of Global Health and Social Medicine, Brigham and Women's Hospital and Harvard Medical School, Boston, MA, United States

**Keywords:** tuberculosis, T cell receptor, CD1b, tetramer, glycolipids, mycobacteria

## Abstract

The non-polymorphic nature of CD1 proteins creates a situation in which T cells with invariant T cell receptors (TCRs), like CD1d-specific NKT cells, are present in all humans. CD1b is an abundant protein on human dendritic cells that presents *M. tuberculosis* (*Mtb*) lipid antigens to T cells. Analysis of T cell clones suggested that semi-invariant TCRs exist in the CD1b system, but their prevalence in humans is not known. Here we used CD1b tetramers loaded with mycolic acid or glucose monomycolate to study polyclonal T cells from 150 Peruvian subjects. We found that CD1b tetramers loaded with mycolic acid or glucose monomycolate antigens stained TRAV1-2^+^ GEM T cells or TRBV4-1^+^ LDN5-like T cells in the majority of subjects tested, at rates ~10-fold lower than NKT cells. Thus, GEM T cells and LDN5-like T cells are a normal part of the human immune system. Unlike prior studies measuring MHC- or CD1b-mediated activation, this large-scale tetramer study found no significant differences in rates of CD1b tetramer-mycobacterial lipid staining of T cells among subjects with *Mtb* exposure, latent *Mtb* infection or active tuberculosis (TB) disease. In all subjects, including “uninfected” subjects, CD1b tetramer^+^ T cells expressed memory markers at high levels. However, among controls with lower mycobacterial antigen exposure in Boston, we found significantly lower frequencies of T cells staining with CD1b tetramers loaded with mycobacterial lipids. These data link CD1b-specific T cell detection to mycobacterial exposure, but not TB disease status, which potentially explains differences in outcomes among CD1-based clinical studies, which used control subjects with low *Mtb* exposure.

## Introduction

During the human immune response to *Mycobacterium tuberculosis* (*Mtb*), MHC-restricted T cells are primed by peptide antigens, and their numbers increase in the blood of infected patients (1). In fact, two of the most common diagnostic tests for *Mtb* infection, intradermal purified protein derivative (PPD) and interferon-γ release assays (IGRA), depend on the reliable and durable expansion of peptide antigen specific T cells in human blood. However, other antigen presentation systems exist that are encoded by non-polymorphic genes. The human CD1 family (CD1a, CD1b, CD1c, CD1d), and MR1 present lipids and metabolites to T cells, respectively (2–4). Because responding T cells are not restricted to the genetic background of the host, such T cells are considered donor unrestricted T cells (DURTs) (5). DURTs are of interest to basic immunologists because they raise new questions about innate function in relation to the use of invariant TCRs, which are seen in NKT cells and MAIT cells that recognize CD1d and MR1, respectively. For clinicians, the non-polymorphic aspect of the CD1 system creates a situation in which any individual might respond to one kind of immunizing antigen, and conserved TCR patterns might allow rapid methods to detect expansion of antigen-specific T cells.

CD1b is expressed at high levels on myeloid dendritic cells in blood and in tissues, and on certain macrophages and other immune cells in the periphery. There is now extensive *in vitro* and molecular evidence for CD1b presentation of mycobacterial lipid antigens to T cells. CD1b presents many mycobacterial lipid antigens, including glucose monomycolate (GMM) and free mycolic acid (MA) to human T cell clones (6, 7). The responding T cell clones show effector functions that are consistent with a role in host protection, including Th1 skewed responses, cytotoxicity toward infected cells, and lack of response to uninfected cells or self-lipids (8–12).

Translating these insights from *in vitro* studies of T cell clones into a broader understanding of the natural polyclonal T cell response *in vivo* in humans represents a major goal of CD1 research. One study of a transgenic mouse expressing a human MA-specific T cell receptor (TCR) and CD1b showed evidence for T cell infiltration into mouse lung and a small but detectable lowering of bacterial counts (2). Thus, organ-specific host response and protection could exist, but is difficult to study in humans given the limitations of *in vitro* T cell activation assays. Human peripheral blood mononuclear cells (PBMC) contain a sample of the highly diverse T cell repertoire in an individual, in which each antigen specificity is represented at low frequency. Several human studies demonstrate that, compared to uninfected individuals, people with latent or active tuberculosis (TB) show increased T cell recognition of mycobacterial lipids, including mannosylphosphodolichol (13), glucose monomycolate (14), mycolic acid (15, 16), sulfoglycolipid (17), and glycerol monomycolate (18).

These studies were conducted using activation assays of T cells measured *ex vivo* using lipid antigens from mycobacteria. Even trace bacterial peptides in lipid preparations made from lipids can provide false positive T cell activation. However, this concern is mitigated by blockade of responses with monoclonal antibodies recognizing CD1, and the concern is eliminated in those studies that use synthetic lipid antigens. Another limitation of activation assays is that low numbers of false positive events can significantly alter the outcome of quantification of low frequency antigen-specific T cells. Also, activation assays are subject to “bystander effects” whereby immune cells that are indirectly activated by cytokines and non-TCR-based mechanisms can cause an overestimate of the number of antigen specific T cells (19).

To bypass certain technical limitations of activation assays, fluorescent CD1b tetramers loaded with GMM or MA can directly detect and capture T cells that express antigen specific TCRs. Human CD1b, CD1a, and CD1c tetramers have been used to demonstrate the existence of CD1-reactive T cells in TB patients (12, 20–23), but systematic comparisons to uninfected controls have not been reported. One potential advantage of tetramers is that the method is not destructive of cells identified, so information on the cell surface phenotype of the antigen-specific T cells can be derived. Also, two TCR-defined populations are known in the CD1b system, which provides a simple method to validate that tetramers are binding to TCRs of interest. For example, Germline-Encoded Mycolyl lipid reactive (GEM) T cells are defined by the expression of nearly invariant TRAV1-2/TRAJ9^+^ TCR α chains and CD4^+^ (24). LDN5-like T cells, named after the clone LDN5, use TRAV17 or TRBV4-1, but have highly variable joining regions and do not seem to preferentially use any particular J segments (25). These two recognized CD1b-reactive T cell populations have never been studied in a large human cohort, and CD1b tetramers in combination with antibodies against TRAV1-2 or TRBV4-1 could detect their prevalence in humans for the first time.

The initial goal of this study was to measure the frequency of CD1b and mycobacterial lipid-reactive T cells using CD1b tetramers in a cohort of 150 human subjects that had either *Mtb* exposure but no documented infection, latent infection, or active TB. The possibility that CD1b-reactive TCRs might be expanded in infected patients is suggested by the behavior of MHC-restricted T cells and prior studies of CD1-reactivity using activation assays discussed above. On the other hand, other T cells recognizing non-polymorphic antigen presenting molecules, like NKT cells and MAIT cells, show no or variable changes when measured in the blood, consistent with a role in local rather than systemic inflammation (26–30). For CD1b-specific T cells, including GEM T cells and LDN5-like T cells, rates of T cells carrying antigen-specific TCRs have not been previously measured on a large scale with tetramers.

## Materials and Methods

### Human Subjects

Subjects with active TB and their household contacts were recruited through Socios En Salud, an affiliate of Partners in Health, based in Lima, Peru. We enrolled 50 patients with culture confirmed pulmonary TB and 100 of their asymptomatic household contacts of whom 50 had positive IGRA tests, as determined by the QuantiFERON TB-Gold In-Tube assay (Qiagen). Fifty subjects were IGRA^−^ so they were defined as “exposed but uninfected”. Participants were at least 14 years old and had a negative HIV serology test. Peripheral blood mononuclear cells (PBMC) were isolated from 50 ml of blood and cryopreserved at 5 × 10^6^ cells per aliquot. The Brigham and Women's Hospital Specimen Bank, Boston, provided de-identified leukoreduction filters from local blood bank donors for PBMC isolation. The Institutional Review Board of the Harvard Faculty of Medicine and Partners Healthcare, and the Institutional Committee of Ethics in Research of the Peruvian Institutes of Health approved this study protocol. All adult study participants and parents or legal guardians of minors had to provide informed consent, while minors provided assent.

### Flow Cytometric Analyses

Pure methoxy MA (C79-87 chain length) was isolated from *Mtb* MA methyl esters by thin layer chromatography, followed by saponification as described (31). CD1b monomers were loaded with *Rhodococcus equi* GMM or *Mtb* methoxy MA as described (12, 23) and tetramerized with streptavidin-phycoerythrin (PE). Cryopreserved samples were thawed at 37°C and were expanded or tested directly after thawing. For expansion, 10^6^ cells were cultured with 25 × 10^6^ irradiated allogeneic PBMC, 5 × 10^6^ irradiated allogeneic Epstein Barr Virus transformed B cells, 30 ng/ml anti-CD3 monoclonal antibody (clone OKT3) for 14–16 days, in the presence of 1 ng/ml interleukin-2 (IL-2). ~ 3 × 10^6^ cells were stained with a “live-dead” fixable blue cell stain (Molecular Probes), then treated with tetramer in staining medium (5% bovine serum albumin and 0.01% sodium azide in PBS) for 10 min at room temperature in the dark, followed by cell surface antibodies for 5 min. Subsequently, cells were treated with unlabeled OKT3 antibody and incubated for 5 min at room temperature, followed by 10 min at 4°C. Cells were fixed in fresh 2% paraformaldehyde (Electron Microscopy Sciences) in PBS for 20 min. The flow cytometry panels and antibodies used for PBMC and for expanded T cells are shown in Supplementary Table 1. Cells were analyzed on a BD LSRFortessa Cell Analyzer equipped with a 355, 405, 488, 561, and 640 nm laser. For each subject identical numbers of events were recorded from each of the three stained samples; the number of recorded events per sample was between 0.9 × 10^6^ and 2.0 × 10^6^.

### Data Analysis and Statistics

Flow cytometry data were analyzed using Flowjo (version 10.4.2). Although PBMCs were collected from 50 subjects in each of the three groups, not every sample passed quality control for cell yield, viability and lack of contamination during expansion. The number of analyzable expanded T cell samples was: 50 from latent *Mtb* infection, 48 from active TB, and 49 from uninfected household contacts. The number of analyzable PBMC samples was: 48 from latent *Mtb* infection, 42 from active TB, and 44 uninfected household contacts. All analyzable samples are listed in Supplementary Table 2. To plot percentages of tetramer^+^ T cells on a log scale, all 0 values were replaced by 0.00005. For analysis of frequencies of GEM and LDN5-like cells, samples with < 10 cells in the CD1b GMM tetramer^+^ gate were excluded from the analysis. Statistical analyses were performed in GraphPad Prism (version 7), except the permutation test, which was performed using SPICE, available at http://exon.niaid.nih.gov/spice (32).

## Results

### Study Design and Validation of Reagents

PBMC were isolated from 50 Peruvian individuals with active TB before the start of anti-TB drug treatment. In addition, 50 IGRA^+^ household contacts with no clinical signs of active TB (latent *Mtb* infection), and 50 IGRA^−^ household contacts (exposed but uninfected) participated in the study (Table 1). Household contacts, as opposed to age-matched controls, were chosen to minimize differences in exposure to other pathogens and environmental microbes among the three groups. Also, this cross-sectional design reduces variables related to differing socioeconomic factors. Demographic characteristics of participants in both arms of the study after removal of unanalyzable samples are shown in Tables 1, 2.

**Table 1:** Evaluable participants in tetramer analysis of expanded T cells.

**Variable**	**Uninfected** **(*n* = 49)**	**Latent** **(*n* = 50)**	**Active tuberculosis** **(*n* = 48)**	***P*-value**
Median age, years (Interquartile range)	27 (21-40)	38.5 (26.25–52)	28.5 (19-43)	Kruskal-Wallis 0.025
Gender, n (%) Male	24 (49)	19 (39.6)	32 (66.7)	χ^2^ 0.02

**Table 2:** Evaluable participants in tetramer analysis from PBMC samples directly *ex-vivo*.

**Variable**	**Uninfected** **(*n* = 44)**	**Latent** **(*n* = 48)**	**Active tuberculosis** **(*n* = 42)**	***P*-value**
Median age, years (Interquartile range)	30.5 (22-41)	38 (25.75–52)	28 (19-40)	Kruskal-Wallis 0.036
Gender, n (%) Male	20 (45.5)	18 (37.5)	27 (64.3)	χ^2^ 0.028

**Figure 1:**
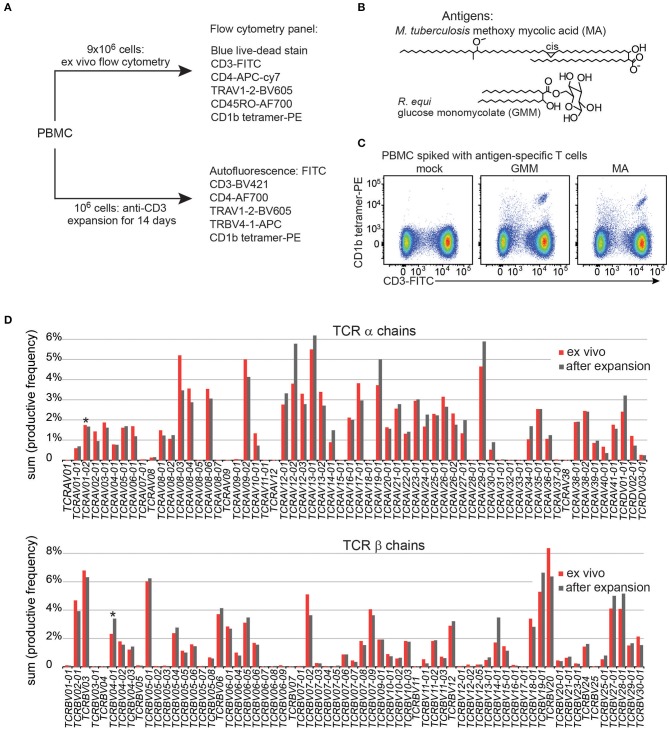
Experimental approach of CD1b tetramer study. **(A)** Flow cytometric study of 150 Peruvian subjects was conducted with two arms, existing of expanded T cells and direct *ex vivo* peripheral blood mononuclear cells (PBMC). **(B)** Chemical structures of natural methoxy mycolic acid from *Mtb* and glucose monomycolate from *Rhodococcus equi*, which were both used to load CD1b tetramers. **(C)** Validation of CD1b tetramers using PBMC from a Boston blood bank donor, spiked with the relevant antigen specific T cell line. **(D)** T cell expansion did not dramatically alter the TCR variable (V) gene usage when compared to PBMC from the same blood donor.

To gain adequate numbers of T cells while also minimizing *in vitro* manipulation, we designed a study with two arms. First, ~ 9 × 10^6^ PBMC from each patient were analyzed directly after thawing to determine frequencies of T cells and measure their activation markers in the *ex vivo* state. Second, to increase yields and generate adequate numbers of highly pure, viable T cells for TCR analyses, an aliquot of 10^6^ PBMC was cultured for one round of stimulation with anti-CD3, typically generating ~ 25 × 10^6^ T cells (Figure 1A). The flow cytometry staining panels for fresh and expanded T cells were similar except that CD45RO was omitted for the expanded T cells because these memory and activation markers are upregulated during *in vitro* stimulation and expansion of T cells. Flow cytometry panels used in both arms of the study contained three CD1b tetramers: mock treated CD1b (CD1b-mock), CD1b-MA, and CD1b-GMM (Figure 1B). The particular forms of MA (*Mtb* methoxymycolate) and GMM (*R. equi* glucose-6-O-monomycolate) were chosen based on prior studies that documented loading onto CD1b and optimal staining of human T cell lines (12, 23). Loading was confirmed for this study by staining PBMC spiked with CD1b-reactive T cell lines (Figure 1C). We used TCR V region specific antibodies to distinguish GEM T cells (TRAV1-2^+^, CD4^+^, CD1b tetramer^+^) from LDN5-like T cells (TRBV4-1^+^, CD1b tetramer^+^) from unclassified CD1b tetramer^+^ cells (Figure 1A).

Large-scale human CD1b tetramer studies had not been carried out previously, so we first validated key aspects of CD1b tetramer reagents, batching issues and the expansion protocol. Preliminary studies of CD1b tetramers loaded with MA or GMM (Figure 1B) on PBMC from a blood bank donor spiked with the GMM-reactive T cell line LDN5 (7) or MA specific C58L (23) showed bright staining with the expected number of cells. Among positive cells, the pattern revealed a positive correlation between anti-CD3 and tetramer in a diagonally shaped pattern, as expected for anti-TCR binding by tetramers (Figure 1C and Supplementary Figure 1). To reduce systematic bias by variations in tetramer batches, we stained equal numbers of subjects from each patient group in every experiment. To evaluate the effects of expansion on the TCR repertoire, we measured frequencies of all TCR α and β V regions, including the characteristic V genes for GEM T cells (TRAV1-2) and LDN5-like T cells (TRBV4-1) using high throughput TCR sequencing of 10^6^ *ex vivo* PBMC and 10^6^ expanded T cells from the same donor. The TCR V gene usage before and after expansion was highly comparable overall (Figure 1D). Considering the variable region gene that defines GEM T cells, TRAV1-2 was 1.7% before expansion and 1.6% after. For LDN5-like T cells, TRBV4-1 was 2.3% before and 3.7% after expansion. Thus, one round of antigen-independent *ex vivo* T cell expansion did not introduce systematic bias in TCR V gene usage.

### Frequency CD1b Tetramer^+^ T Cells

**Figure 2:**
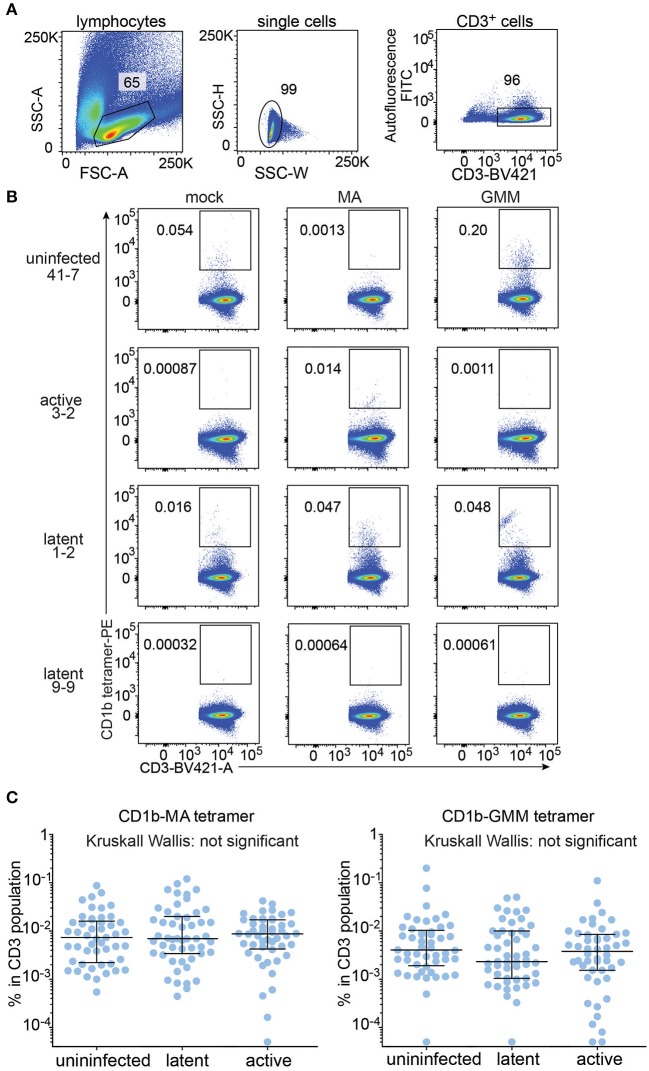
Quantification of CD1b-mycolic acid and CD1b-glucose monomycolate tetramer^+^ T cells in a Peruvian TB cohort. **(A)** Expanded peripheral blood mononuclear cells were gated based on forward and side scatter, as well as CD3 expression and low autofluorescence, which was determined in the FITC channel. **(B)** Representative flow cytometry plots of four of the 150 members of the cohort after staining with CD1b-mock, CD1b-MA, and CD1b-GMM tetramers after pre-gating as shown in **(A)**. Numbers indicate percent cells in gate. **(C)** Frequencies of tetramer^+^ T cells among all subjects of the Peruvian cohort, analyzed by TB disease status. Medians and interquartile ranges of tetramer^+^ T cells are depicted as a percent of total CD3^+^ cells.

All analyzable expanded samples (*n* = 147) showed low autofluorescence (Figure 2A) and low but detectable background staining with mock treated CD1b tetramers (Figure 2B, left column and Supplementary Figure 2). CD1b-mock tetramers contain phospholipids from the cellular expression system (33, 34) so CD1b-mock staining represents specific staining of phospholipid-reactive TCRs, broadly cross-reactive TCRs, and TCRs that recognize CD1b alone, rather than non-specific adherence to T cells. Because the three tetramers were labeled with phycoerythrin (PE), we could set identical gates for all tetramers for every member of the cohort, with four examples shown in detail (Figure 2B). Compared to the median frequency of CD1b-mock tetramer^+^ T cells (0.0022%), the median frequencies of CD1b-GMM tetramer^+^ T cells (0.0034%) and CD1b-MA tetramer^+^ T cells (0.0073%) in the cohort of 147 patients provide evidence for broad, albeit low absolute levels for CD1b reactivity to these lipids in humans. As a point of reference, the median frequency of human CD1d-reactive NKT cells is 0.03% (35), so the CD1b tetramer^+^ T cell frequencies here are ~10-fold lower than NKT cells. Comparing the median tetramer staining rate among the three groups based on TB disease status, the frequencies of CD1b-MA and CD1b-GMM tetramer^+^ blood T cells did not significantly differ among uninfected subjects, latently infected subjects and TB patients as determined by the Kruskall-Wallis test (Figure 2C). Thus, CD1b-restricted cells do not behave like MHC-restricted cells by increasing in frequency in the blood after infection by *Mtb*.

### Subpopulations of CD1b-GMM Specific T Cells

**Figure 3:**
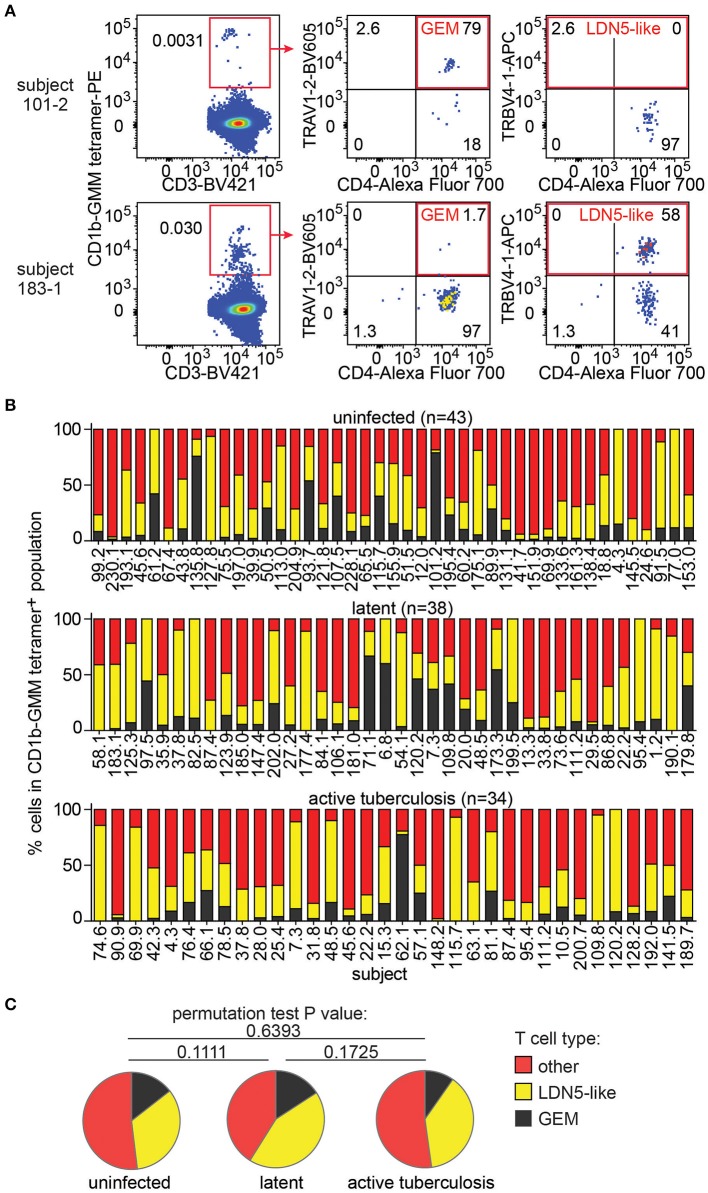
GEM T cells and LDN5-like T cells are simultaneously present in the T cell repertoire in Peruvian subjects. **(A)** After pre-gating as in Figure 2A, detection of GEM T cells and LDN5-like T cells was based on CD1b-GMM tetramer and antibodies against CD4 and TCR variable segments TRAV1-2 or TRBV4-1. **(B)** Quantification of the proportion of frequencies of GEM T cells, LDN5-like T cells and other CD1b-GMM tetramer^+^ T cells that lack defining variable region segments in active TB patients (*n* = 34), IGRA^+^ (latent infection) (*n* = 38) household contacts and IGRA^−^ (uninfected) (*n* = 43) household contacts. **(C)** Median proportions of T cell subsets of CD1b-GMM tetramer^+^ T cells analyzed according to TB disease status are shown.

Next, we took advantage of the combination of tetramer and anti-TCR V region antibodies to detect LDN5-like T cells and GEM T cells in the expanded samples. LDN5-like T cells were described as a TCR motif based on study of six T cell clones studied outside the context of TB (25). Polyclonal GEM T cells have been previously detected in small numbers of subjects (24, 36), but their prevalence among humans, including TB patients, remained unknown. In 147 analyzable samples, we first gated CD1b-GMM tetramers^+^ T cells to identify samples with 10 or more cells in the gate (*n* = 115, Supplementary Table 2). From these, we quantified TRAV1-2^+^CD4^+^ T cells to define GEM T cells and TRBV4-1^+^ to define LDN5-like T cells as illustrated in two representative patients (Figure 3A). The remaining CD1b-GMM tetramer^+^ T cells did not fit a known TCR motif and were considered to be diverse CD1b-GMM-specific T cells.

We found that these three T cell populations typically co-exist in each sample studied (Figure 3B), indicating that CD1b-reactive TCR patterns are broadly conserved in humans. Across all samples, within the CD1b-GMM tetramer^+^ gate, the relative frequency of GEM T cells (median 8%) is less than LDN5-like T cells (median 29%), which is less than diverse CD1b-GMM-specific T cells (median 50%). Next, we examined frequencies according to TB infection status and found that the distribution of GEM T cells, LDN5-like T cells and diverse T cells within the CD1b-GMM tetramer^+^ gate was not significantly different among the uninfected, latently infected and active TB groups (Figure 3C).

### CD1b Tetramer^+^ T Cells Express CD45RO

**Figure 4:**
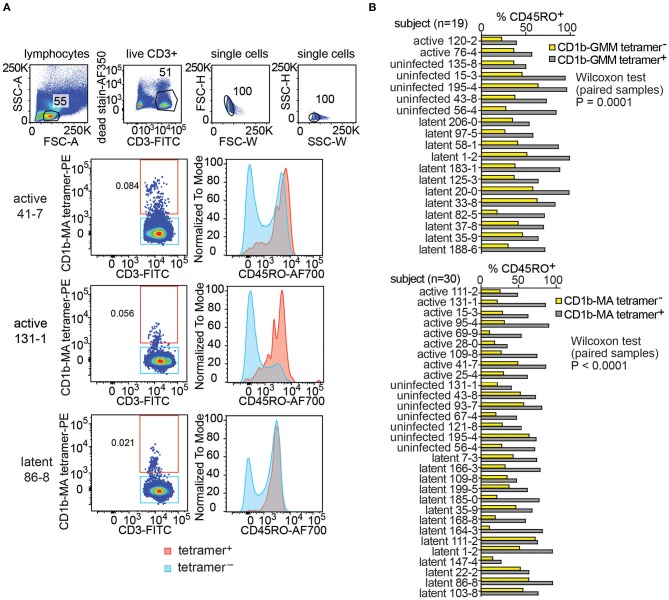
Expression of CD45RO on fresh PBMCs from a Peruvian cohort. Using the indicated pre-gating strategy for fresh PBMCs **(A)**, flow cytometry plots of CD45RO expression among CD1b tetramer^+^ T cells and CD1b tetramer^−^ T cells of three members of the Peruvian cohort. **(B)** The proportion of CD45RO^+^ among tetramer^+^ and tetramer^−^ T cells in the analyzable PBMC samples of the Peruvian cohort.

Little is known about the origin and maintenance of CD1b-reactive T cells. Such cells might be primed by infection. Alternatively, they might be broadly activated and pre-primed *in vivo* in the absence of infection, like NKT cells, MAIT cells and other innate T cells that recognize non-polymorphic antigen presenting molecules (37). Insight in their activation state can be obtained from CD45RO expression, which is elevated in cells that show properties of immunological memory. As expected, CD1b-MA tetramer^−^ T cell populations showed the typical bimodal distribution of CD45RO expression on T cells, separating two large pools of memory (CD45RO^+^) and naive T cells (CD45RO^−^). This pattern is shown for 3 representative subjects in Figure 4A. In contrast, CD1b-MA tetramer^+^ T cells expressed higher levels of CD45RO than tetramer^−^ T cells. In two of the first three subjects studied, CD45RO was nearly uniformly positive in CD1b-MA tetramer+ cells (Figure 4A).

This striking finding led to broader analysis of CD1b-GMM tetramer^+^ and CD1b-MA tetramer^+^ cells in 49 evaluable subjects with clearly detectable CD1b-GMM tetramer^+^ or CD1b-MA tetramer^+^ pattern (Figure 4B and Supplementary Table 2). In all 19 subjects studied, the frequency of CD45RO^+^ cells was higher among CD1b-GMM tetramer^+^ cells as compared to CD1b-GMM tetramer^−^ T cells, a difference that was highly statistically significant (*P* = 0.0001). Similarly, for CD1b-MA tetramer stained cells, CD45RO was higher in tetramer^+^ cells compared to tetramer^−^ cells among all 30 patients studied (*P* < 0.0001). Across all evaluable samples, 73 ± 19 % of the CD1b-GMM tetramer^+^ T cells and 67 ± 17 % of the CD1b-MA tetramer^+^ T cells were CD45RO^+^.

Given the usefulness of CD45RO in predicting a memory phenotype, the higher expression of this marker among CD1b tetramer^+^ T cells among all donors tested and the statistically significant result for both antigens, these results suggested prior *in vivo* activation of such cells. While *Mtb* infection might be the source of such prior activation, we had no ready explanation for the high CD45RO expression in the “uninfected” group. Such cells might have induction of memory markers by a stimulus other than *Mtb*. Alternatively, given that these subjects were living with an active TB patient in an endemic area, their IGRA^−^ status might be inadequate evidence for lack of prior infection. Indeed, several recent studies have introduced the concept that highly exposed individuals might have immune response to infecting *Mtb*, even if conventional clinical tests like IGRA are not converted (38–41).

### CD1b Tetramer Staining for Low Exposure Subjects

Considering possible explanations for the universal appearance of memory markers, we realized that all subjects, including “uninfected” subjects in Lima were exposed to mycobacteria by the active TB patient in their household. Also, BCG vaccination has 80% coverage in Peru [www.bcgatlas.org/]. Further, IGRA^−^ subjects in Lima might have been exposed to *Mtb* in the community or were possibly exposed to non-tuberculous mycobacteria. BCG is not routinely administered in the USA where TB incidence of 3/100,000 is more than 5-fold lower than in Peru (42) [www.who.int/tb/publications/global_report/en/]. If activation of CD1b-lipid-specific T cells in the Peruvian cohort were a result of high exposure, but without infection, the healthy blood bank donors in the USA should have lower numbers of CD1b-lipid tetramer^+^CD45RO^+^ than “uninfected” but highly exposed subjects in Peru.

**Figure 5:**
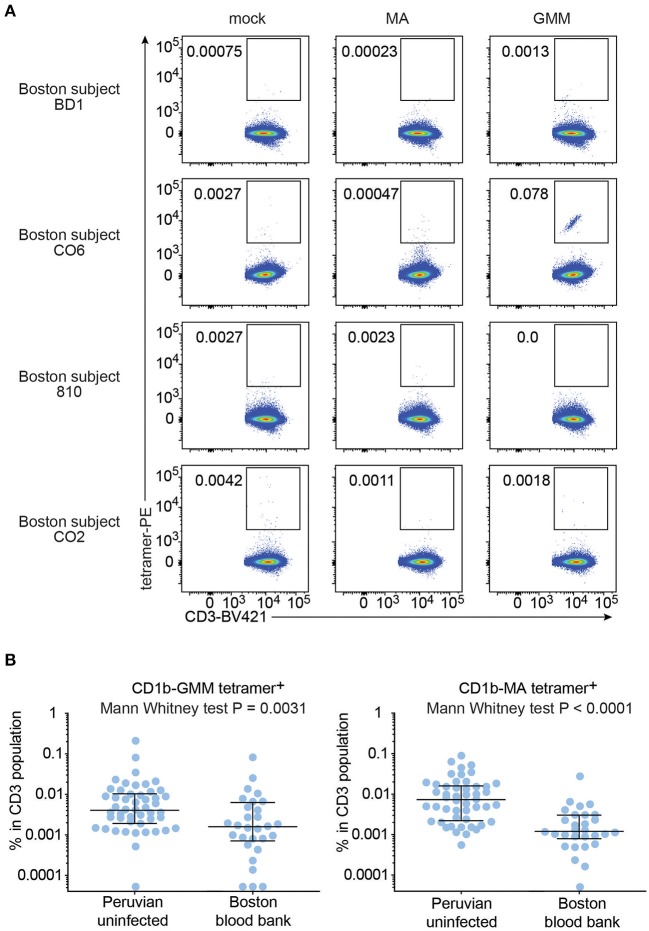
Frequencies of CD1b-MA and CD1b-GMM tetramer^+^ T cells among uninfected Peruvians and Boston blood bank donors. **(A)** Representative flow cytometry plots of CD1b-mock, CD1b-MA, and CD1b-GMM tetramer staining among CD3^+^ T cells from expanded PBMC derived from 29 Boston blood bank donors analyzed using the same protocol as for the Peruvian cohort. **(B)** Frequencies of CD1b-MA and CD1b-GMM tetramer^+^ T cells among uninfected Peruvian household contacts of TB patients and Boston blood bank donors.

To test this hypothesis, we recruited 29 blood bank donors from Boston for comparison to the Peruvian cohort. Boston blood bank donors showed very low or undetectable rates of CD1b-MA and CD1b-GMM tetramer^+^ T cells compared to CD1b-mock tetramer^+^ T cells (Figure 5), with the exception of a population of brightly staining cells staining with CD1b-GMM in subject C06 (Figure 5A). Although this analysis used the same methods, reagents, and flow cytometers in the same facility, Boston and Peruvian donors were not analyzed at the same time, raising the possibility of batch effects or changes in reagents over time. In parallel with processing the Boston donors, we thawed 6 aliquots of PBMC from previously tested members of the Peruvian cohort and performed tetramer analysis for comparison between two different time points (Supplementary Figure 3). The results obtained during the initial series of experiments were not significantly different from the results from the same samples studied during the testing of the Boston donors (*P* = 0.11). Thus, reanalysis at a different time point was unlikely to be the cause of substantial bias. After analysis of 29 Boston donors, the mean frequencies of CD1b-GMM-tetramer^+^ (*P* = 0.0031) and CD1b-MA-tetramer^+^ T cells (*P* < 0.0001) were significantly lower compared to the Peruvian “uninfected” group. The frequencies in Boston donors were 3.5 times lower for GMM-tetramer^+^ T cells and 6.3 times lower for CD1b-MA^+^ T cells. This result is consistent with expansion and selective upregulation of memory markers on CD1b-lipid tetramer^+^ T cells in all Peruvian donors, regardless of *Mtb* infection state.

Even though on average, Boston donors showed lower frequencies of tetramer^+^ T cells than Peruvian “uninfected” subjects, some individual Boston donors' frequencies were rather high. Analysis of the CD45RO expression in the Boston donors with the highest frequency of tetramer^+^ T cells showed an enrichment of CD45RO expression among tetramer^+^ T cells, comparable to what was seen among tetramer^+^ T cells from Peruvian donors (Supplementary Figure 4).

## Discussion

These data indicate that CD1b tetramer-based studies can be carried out on T cells from large human cohorts with low rates of false positive staining and good reproducibility among batches. A key finding is that GEM T cells and LDN5-like T cells are detectable in most humans. Whereas NKT cells, MAIT cells and GEM T cells show nearly invariant TCR α chains, LDN5-like cells show conservation in the TCR β chain outside the CDR3. Looking outside CD1d and MR1 systems, finding CD1b-lipid tetramer^+^TRAV1-2^+^ and CD1b-lipid tetramer^+^TRBV4-1^+^ T cells in most humans now expands prior molecular studies to show that polyclonal GEM T cells and LDN5-like T cells are normal components of the human T cell repertoire.

Our initial hypothesis that CD1b-GMM and CD1b-MA-specific T cells might expand in numbers in the peripheral blood during latent or acute infection with *Mtb* was not confirmed in a Peruvian cohort of 150 people. This finding led us to consider the possibility that the uninfected group, which consisted of IGRA^−^, but heavily exposed Peruvians that formed our negative control population, was not the optimal negative control for this experiment. An emerging theme in clinical research on TB is that IFN-γ based diagnostics are potentially too narrow to detect all patients that have been infected by *Mtb*, and that other criteria such as Th2 T cell response or immunoglobulin responses may be relevant in highly exposed people (38–41). Therefore, it is plausible that CD1b-GMM and CD1b-MA-specific T cells increased in number after exposure to mycobacteria, even if exposure did not lead to conversion of the peptide antigen-based IGRA test.

Consistent with this hypothesis, IGRA^−^ members of the Peruvian cohort showed markedly higher frequencies of CD1b tetramer staining as compared with a cohort consisting of Boston blood bank donors, who have lower *Mtb* exposure and BCG vaccination rates. However, other factors may play a role, including genetic and environmental differences between the two cohorts. Therefore, further work is needed to isolate and identify the specific effects of exposure on CD1b tetramer staining rates and CD45RO expression. Despite this limitation, the difference in CD1b tetramer staining rates between these two populations is high in absolute terms and is highly statistically significant.

Further, in considering whether latent or active *Mtb* infection correlates with expansion of CD1-reactive T cells, several studies have now shown clearly differing outcomes, inviting consideration of differences in the human populations and detection methods used. For example, many studies have found increases in CD1b- or CD1c-dependent T cell responses against mycobacterial lipids in latent or active TB patients vs. controls using activation-based assays that measure cytokines. Such positive findings have been seen in small (13, 14) medium (15, 16) and large studies (17, 18). In contrast, this large-scale tetramer study, as well as smaller studies that did (36) or did not (11) use CD1 tetramers in South African cohorts, found no differences among highly exposed groups that differed in their IGRA status.

One possible explanation for the differences among study outcomes is that activation-based assays and tetramer assays are detecting different types of T cells. For example, activation-based assays might detect T cells activated secondarily by cytokines rather than CD1-specific TCR. In contrast, tetramer studies measure CD1b-lipid specific TCRs, but are subject to other technical limitations. For example, TCRs need to have a minimum affinity to stain with tetramer, so TCRs below that threshold will be missed, but can be functional in a cytokine release assay.

A second possibility, suggested by our data, is that negative control groups considered “uninfected” have differing rates of response based on differing antigen exposure through BCG vaccination, undetected *Mtb* infection, or *Mtb* exposure without infection. South Africa, like Peru, is an area with high coverage of BCG vaccination and high TB incidence. Studies reported from South Africa and Peru used community-based household contacts as “uninfected controls.” Thus, negative control populations likely have relatively high antigen exposure that is more similar to that of active TB and latently infected subjects. In contrast, studies that found higher T cell responses in latent or active patients, including studies reporting IFN-γ release after stimulation with CD1b-MA (15), CD1b-GMM (14), CD1b-sulfoglycolipid (17), CD1c-phosphomycoketide (13), and CD1b-glycerol monomycolate (18), generally used “uninfected” controls that did not come from a household with a TB case or high exposure community. Thus, the choice of negative control population with defined exposure characteristics now emerges as being crucially important for the detection of differences in frequencies of *Mtb* lipid-specific T cells. Beyond these new considerations of study design and regardless of differing technical approaches, all studies, including the current one, are consistent with the idea that CD1b-mediated responses are part of a first line response against exposure to mycobacteria.

## Data Availability Statement

The datasets generated for this study are available on request to the corresponding author.

## Ethics Statement

The Institutional Review Board of the Harvard Faculty of Medicine and Partners Healthcare, and the Institutional Committee of Ethics in Research of the Peruvian Institutes of Health approved this study protocol. All adult study participants and parents or legal guardians of minors had to provide written informed consent, while minors provided assent.

## Author Contributions

KL, SI, SS, JR, and TO performed experiments. JJ, RC, and LL contributed unique reagents. MM, DM, and IV designed the study. IV wrote the manuscript with input from all authors.

### Conflict of Interest

The authors declare that the research was conducted in the absence of any commercial or financial relationships that could be construed as a potential conflict of interest.
