# Supplementary material for: CD1b Tetramers Broadly Detect T Cells That Correlate With Mycobacterial Exposure but Not Tuberculosis Disease State
Source: Front Immunol. 2020 Feb 14;11:199. doi: 10.3389/fimmu.2020.00199 (PMC7033476; doi:10.3389/fimmu.2020.00199)
Supplement: Supplementary file 1 [file Table_1.pdf]

Supplementary Table 1: List of flow cytometry antibodies for ex vivo PBMC (A) and expanded T cells (B)

A)

Fluorochrome	Antigen	Clone	Catalogue no.	Supplier
AlexaFluor350	Viability	NA	L23105	Molecular Probes
PE	CD1b tetramer	NA	NA	NIH
FITC	CD3	SK7	340542	BD
Brilliant Violet 605	TRAV1-2	3C10	351720	Biolegend
APC-Cy7	CD4	RPA-T4	557871	BD
AlexaFluor700	CD45RO	UCHL1	561136	BD

B)

Fluorochrome	Antigen	Clone	Catalogue no.	Supplier
BV421	CD3	UCHT1	300434	Biolegend
AlexaFluor700	CD4	RPA-T4	300526	Biolegend
Brilliant Violet 605	TRAV1-2	3C10	351720	Biolegend
APC	TRBV4-1	REA871	130-114-242	Miltenyi
PE	CD1b tetramer	NA	NA	NIH
