# Supplementary material for: CD1b Tetramers Broadly Detect T Cells That Correlate With Mycobacterial Exposure but Not Tuberculosis Disease State
Source: Front Immunol. 2020 Feb 14;11:199. doi: 10.3389/fimmu.2020.00199 (PMC7033476; doi:10.3389/fimmu.2020.00199)
Supplement: Supplementary file 3 [file Image_1.pdf]

Supplementary Figure 1

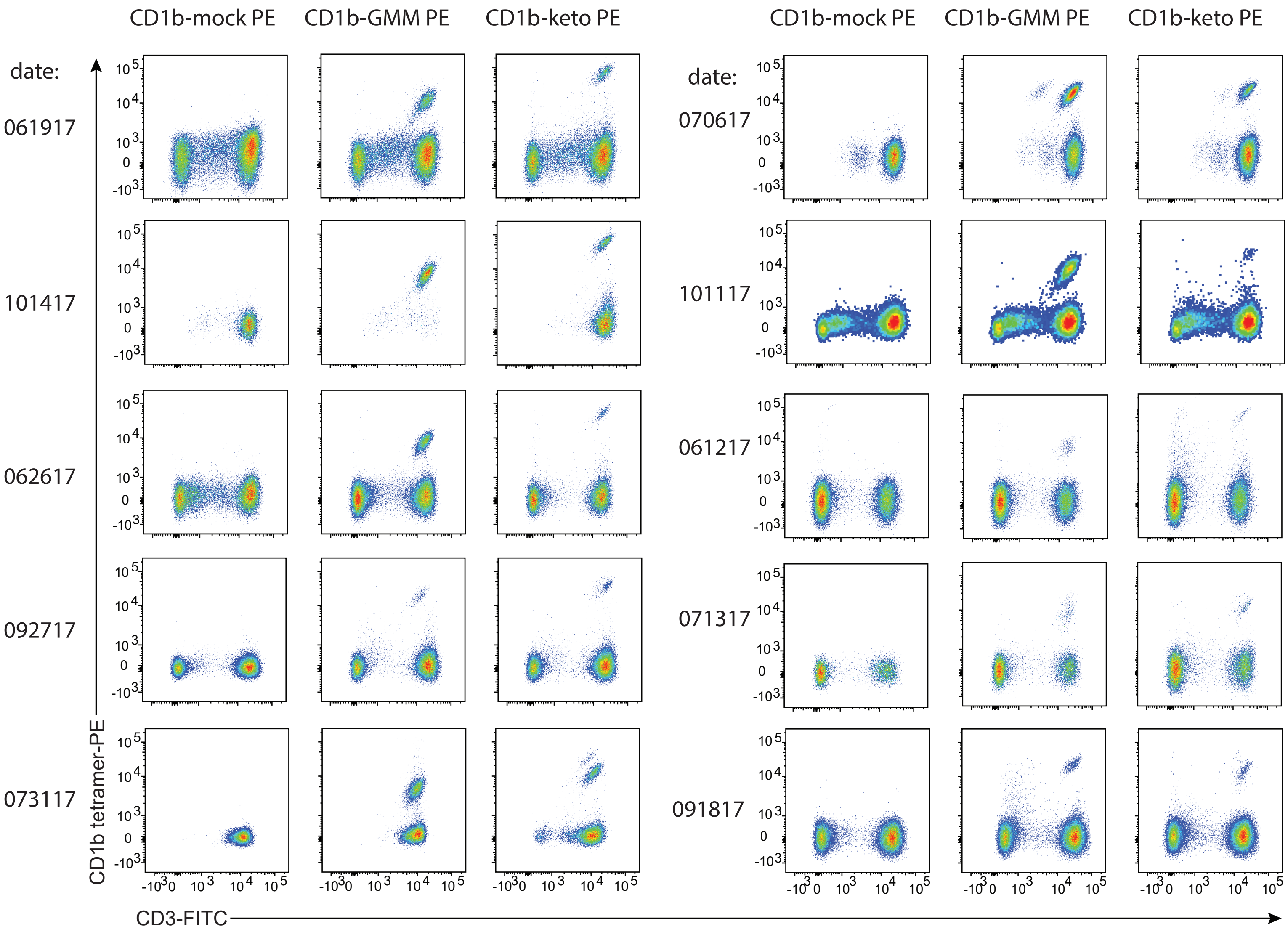

Supplementary Figure 1. Validation of different batches of CD1b tetramers.
Each batch of CD1b tetramers was tested using PBMC from a random Boston donor spiked with positive control cell lines LDN5 (for CD1b-GMM tetramers) or C58L (for CD1b-MA tetramers). Cells were pre-gated based on forward scatter and side scatter and exclusion of multiplets. On October 24, the cell lines were used without mixing into PBMC, and on July 31 the two positive control cell lines were mixed with each other without mixing into PBMC.
