# Supplementary material for: CD1b Tetramers Broadly Detect T Cells That Correlate With Mycobacterial Exposure but Not Tuberculosis Disease State
Source: Front Immunol. 2020 Feb 14;11:199. doi: 10.3389/fimmu.2020.00199 (PMC7033476; doi:10.3389/fimmu.2020.00199)
Supplement: Supplementary file 4 [file Image_2.pdf]

Supplementary Figure 2

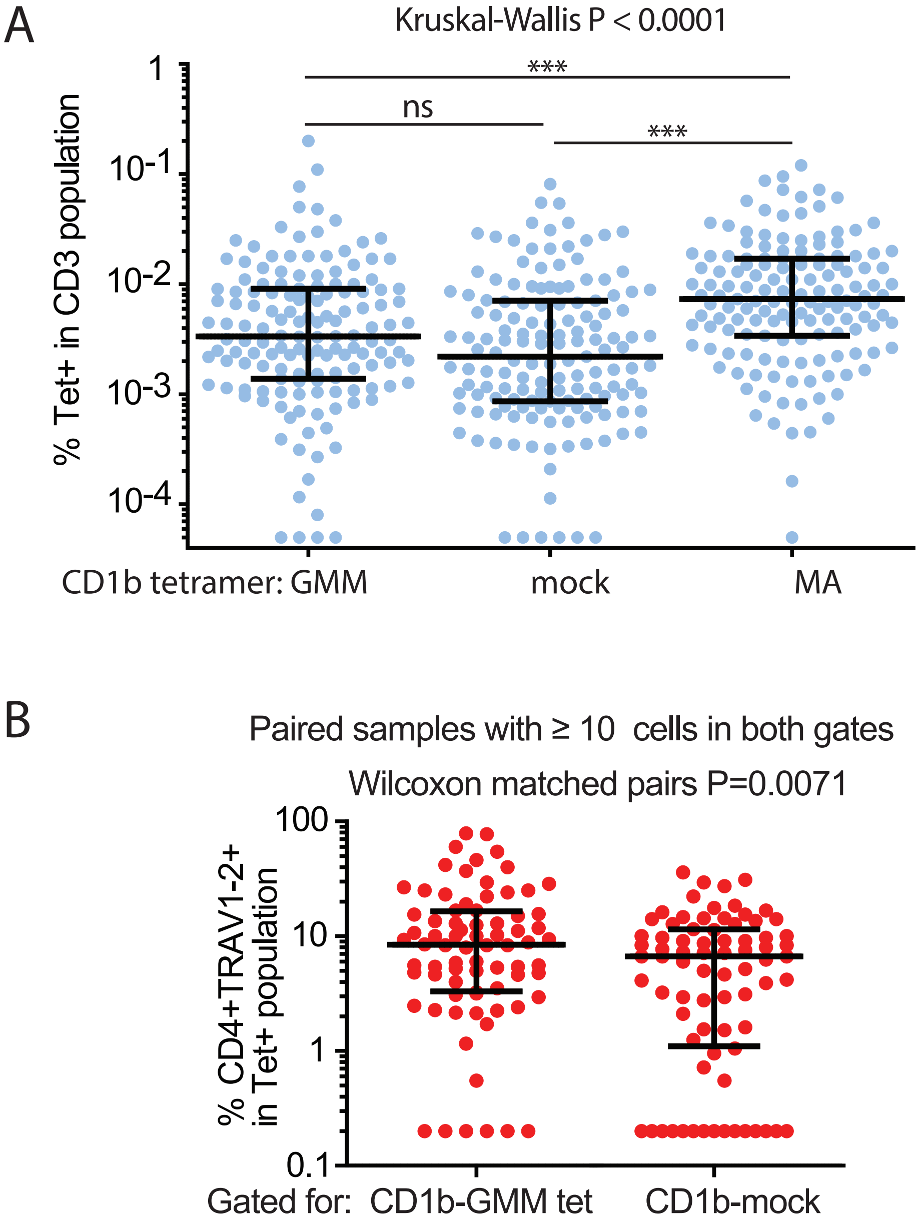

Supplementary Figure 2. Quantification of CD1b tetramer staining in a Peruvian TB cohort. Expanded peripheral blood mononuclear cells were gated based on forward and side scatter, CD3 expression and low autofluorescence, which was determined in the FITC channel, as in Figure 2a. (A) Frequencies of tetramer+ T cells among all subjects of the Peruvian cohort. Medians and interquartile ranges of tetramer+ T cells are depicted as a percent of total CD3+ cells. (B) For subjects that had ≥10 cells in their CD1b-GMM and CD1b-mock gate, the frequency of CD4+TRAV1-2+ cells is shown.
