# Supplementary material for: CD1b Tetramers Broadly Detect T Cells That Correlate With Mycobacterial Exposure but Not Tuberculosis Disease State
Source: Front Immunol. 2020 Feb 14;11:199. doi: 10.3389/fimmu.2020.00199 (PMC7033476; doi:10.3389/fimmu.2020.00199)
Supplement: Supplementary file 5 [file Image_3.pdf]

Supplementary Figure 3

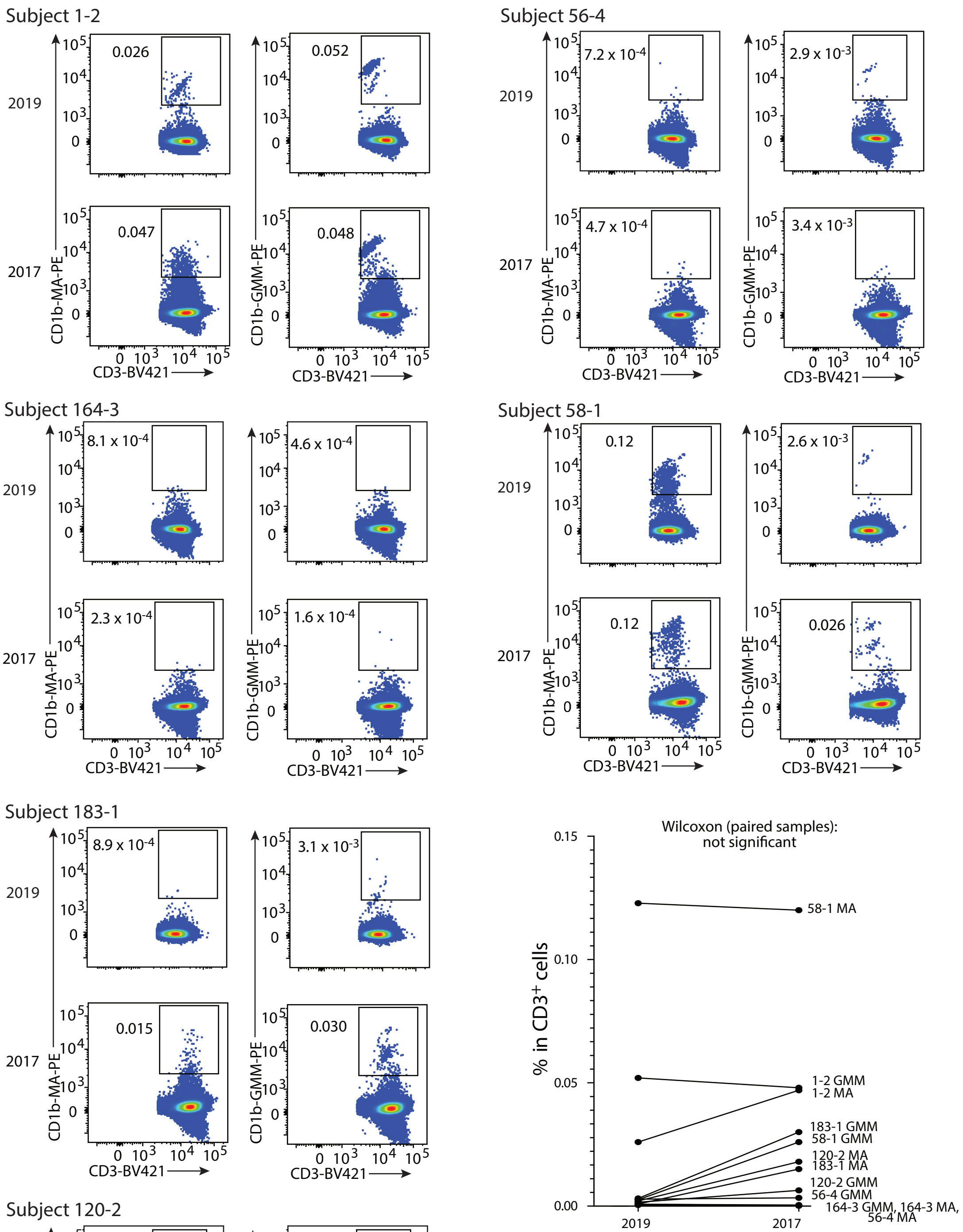

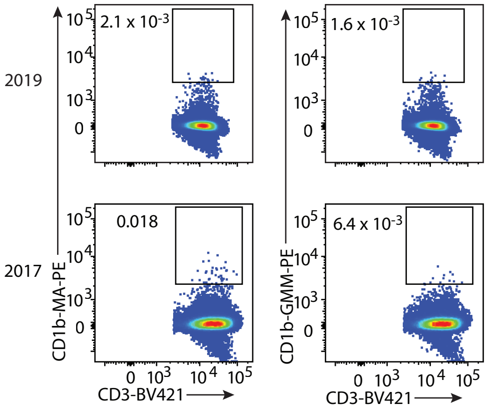

Supplementary Figure 3. Comparison of flow cytometry experiments in 2017 and 2019.
In 2019, an aliquot of PBMC from the same blood draw that was used in 2017, was thawed and stimulated using the same protocol as in 2017. Flow cytometry was performed using the same protocol, but with a different batch of tetramers. For each of the six members of the Peruvian cohort the results obtained in 2017 were compared to the results obtained in 2019. Statistical significance was determined by the Wilcoxon test for paired samples.
