# Supplementary material for: CD1b Tetramers Broadly Detect T Cells That Correlate With Mycobacterial Exposure but Not Tuberculosis Disease State
Source: Front Immunol. 2020 Feb 14;11:199. doi: 10.3389/fimmu.2020.00199 (PMC7033476; doi:10.3389/fimmu.2020.00199)
Supplement: Supplementary file 6 [file Image_4.pdf]

Supplementary Figure 4

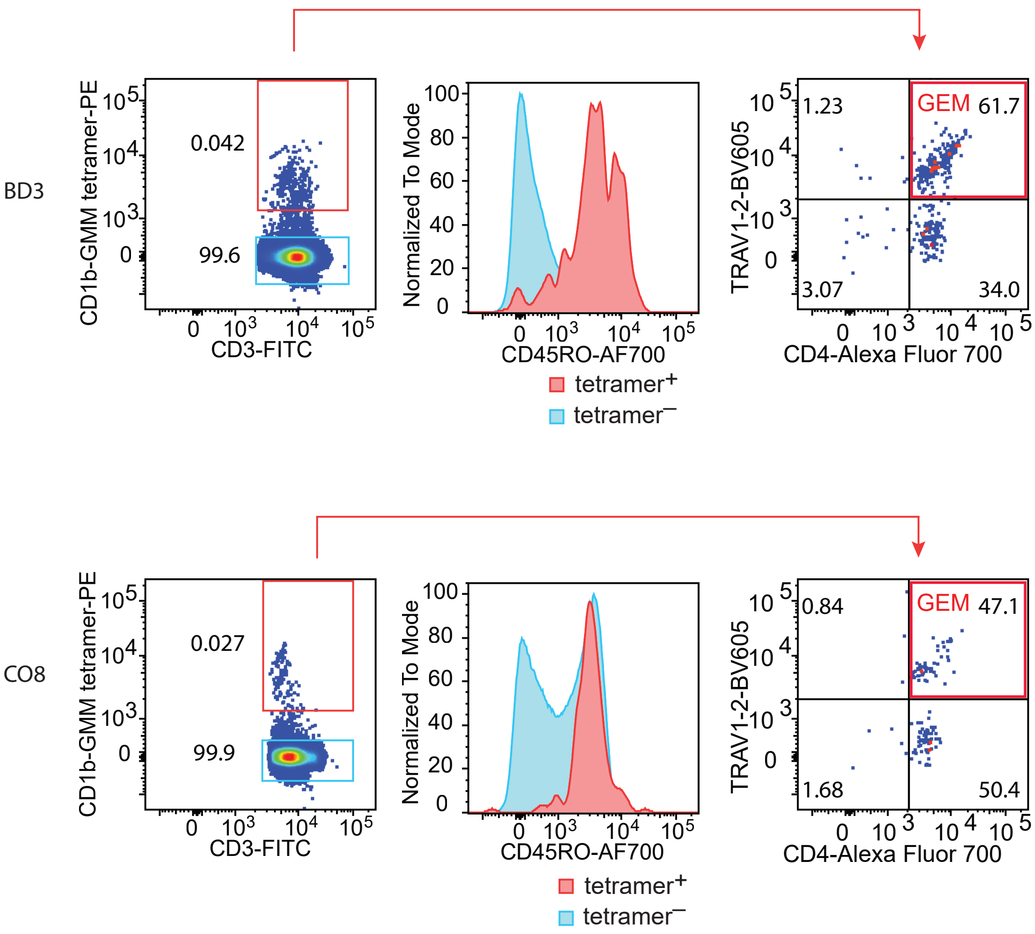

Supplementary Figure 4. Flow cytometry plots of CD45RO expression by CD1b tetramer+ T cells and CD1b tetramer– T cells of two members of the Boston cohort that were selected for having among the highest CD1b-GMM tetramer+ T cell frequencies.
